# Social Competence and Peer Social Acceptance: Evaluating Effects of an Educational Intervention in Adolescents

**DOI:** 10.3389/fpsyg.2020.01305

**Published:** 2020-06-11

**Authors:** Pablo Luna, Jerónimo Guerrero, Débora Rodrigo-Ruiz, Lidia Losada, Javier Cejudo

**Affiliations:** ^1^Department of Psychology, Faculty of Education, University of Castilla–La Mancha, Ciudad Real, Spain; ^2^Department of Physical Education, Instituto de Enseñanza Secundaria (IES) Lazarillo de Tormes, Toledo, Spain; ^3^Department of Research and Diagnostic Methods in Education II, Faculty of Education, International University of La Rioja (UNIR), Logroño, Spain; ^4^Faculty of Education, National University of Distance Education (UNED), Madrid, Spain

**Keywords:** quality physical education, sport education model, social competence, peer social acceptance, gender, adolescents

## Abstract

This study aims to evaluate the impact of an educational intervention on social competence and social acceptance among adolescents. The participants were 106 adolescents aged 12–15 years (*M* = 13.41 years; *SD* = 0.81 years). Participants were randomly assigned to the control group (*n* = 44) and an experimental group (*n* = 69). In the experimental group, an intervention based on the Sport Education Model (SEM) was applied. While in the control group, an intervention based on the Traditional Model of Direct Instruction (TM-DI) was carried out. An experimental design with repeated pretest and posttest measurements was developed. The Adolescent Multidimensional Social Competence Questionnaire (AMSC-Q) was used to assess social competence. The Guess Who (GW4) questionnaire was used to assess social acceptance (SA) among peers. The preliminary results showed that the intervention based on the SEM (experimental group) promoted more significant improvements in some indicators of social competence and social acceptance among peers than those obtained with the TM-DI (control group). The results confirm a similar impact of the intervention between boys and girls. These preliminary results suggest the potential of the Sport Education Model with adolescents.

## Introduction

Quality education requires attending to cognitive and affective-social dimensions that facilitate the physical and psychosocial development of students (United Nations Educational Scientific and Cultural Organization [UNESCO], 2015). The importance of the affective-social dimension in the success of the teaching and learning process in the educational context are issues that are arousing great interest in research (United Nations Educational Scientific and Cultural Organization [UNESCO], 2015; Franco et al., 2017; Méndez-Giménez et al., 2018). Accordingly, the teaching and learning process has an individual and social aspect (Franco et al., 2017), where the search for social objectives can boost school achievement (Elliot et al., 2006; Cecchini-Estrada et al., 2011). To increase this success in the educational context, it is not only necessary to promote cognitive skills, but also to strengthen socio-emotional skills (Greenberg et al., 2003; Payton et al., 2008; Domitrovich et al., 2017; Taylor et al., 2017). Likewise, socio-emotional skills promote interpersonal relationships between students and other educational agents involved (Garn et al., 2011; Bessa et al., 2019; Kao, 2019). Therefore, it is relevant to promote optimal educational and motivational climates in educational contexts that favor a positive psychosocial adjustment and integral development of the student personality (Bisquerra et al., 2015).

The interest in the educational context for the social and emotional dimension, added to the promotion of satisfactory interpersonal skills (being and feeling accepted) (Zhang et al., 2014) have highlighted that social behavior plays an essential role in the abilities of students, especially in adolescents (Gómez-Ortiz et al., 2017) favoring school success (Cappadocia and Weiss, 2011). It has been recognized that social competence is an inclusive, evaluative, and multidimensional construct (e.g., socio-emotional skills; emotional regulation; prosocial behavior; ability to adapt normatively; social adjustment or perceived effectiveness in social interactions) that cannot be understood from a unilateral perspective (Dirks et al., 2007; Santos et al., 2013; Losada, 2018).

Gómez-Ortiz et al. (2019) define social competence as the effectiveness in social interaction, which arises from the use of socio-emotional skills to achieve personal goals over time and in different situations. In this way, social competence encompasses a series of cognitive, social, and emotional abilities of the individual, to manage the interpersonal relationships that occur in different contexts, favoring healthier relationships among others (Del Prette and Del Prette, 2005). Gresham (1988) divides social competence into the following elements: (1) adaptive behavior (physical and language development, academic competencies and independent functional skills); (2) interpersonal behaviors (cooperative and play behaviors; conversation and regulatory acceptance skills); (3) self-perceived behaviors (of oneself: expressing ethical and positive feelings and behaviors); and (4) behaviors toward homework (attention, task resolution, and individual work).

Thereby, social competence is related to the adjustment to the demands of the school environment, interpersonal relationships, emotional health and acceptance among peers (Losada et al., 2017). Also, it would be pertinent to examine and evaluate, through programs or interventions, the impact of this competence or interpersonal skills in the educational context (Gómez-Ortiz et al., 2017, 2019; Losada et al., 2017), especially in adolescent students, because it is a period of maturation and sensitive adaptation (typical transitions of this stage) for personal, social and emotional development (Gómez-Ortiz et al., 2017, 2019; Bessa et al., 2019). Therefore, social competence plays a vital role in the educational process, since it is necessary to favor positive and quality learning (Del Prette and Del Prette, 2005; Elijah and Madeira, 2013).

A primary objective in the educational context is to promote healthy lifestyles (World Health Organization [WHO], 2016), active and participatory (Pate and Dowda, 2019). Accordingly, physical education, within the framework of a Physical Education of quality reinforcing prosocial practices (United Nations Educational Scientific and Cultural Organization [UNESCO], 2015) is instrumentalized as an effective subject to favor an integral commitment of students (Whitehead, 2010; Escalié et al., 2019) by positively developing their cognitive, affective, physical and social spheres (Mitchell and Hutchinson, 2003; Kao, 2019; Sierra-Díaz et al., 2019). The Association for Physical Education [afPE] (2015) states that quality Physical Education acts as a starting point for a commitment to physical activity and sport throughout life. Thus, this subject provides students with active, cooperative and practical resources (Girard et al., 2019) that improve experiences in the school environment (Kohl and Cook, 2013; Sierra-Díaz et al., 2019). Similarly, it encourages students to develop personal and social skills in a real environment that in other subjects would be more complex to teach (Hellison, 2011).

Physical Education is recognized for playing a relevant role in the acquisition of values and competences that contribute to the personal and socio-emotional development of students (Bessa et al., 2019). Thereby, some authors point out that through adequately structured and planned interventions, it could contribute to the social development of students in the subject of Physical Education (Eldar, 2008; Unlu et al., 2011; Gil-Madrona et al., 2019; Sierra-Díaz et al., 2019). Physical Education offers the student a meaningful learning experience driven by the development of social skills (interpersonal interactions, tolerance, and respect) (Cronin et al., 2018; Kao, 2019); social responsibility adherence and team cohesion: group affiliation or identity, cooperative work (Brinkley et al., 2017; Cronin et al., 2018); and reinforcement of the development of social cognition (Bailey, 2006). Therefore, quality Physical Education (United Nations Educational Scientific and Cultural Organization [UNESCO], 2015) will be a crucial ally in the educational context, to promote positive environments in the development of prosocial behaviors (Mayfield et al., 2017), as long as they are promoted in active, participatory and motivating contexts (Shields et al., 2018).

Consequently, it is relevant in the educational context to configure a path of methodological renewal that evolves, as established by United Nations Educational Scientific and Cultural Organization [UNESCO] (2015), toward a quality Physical Education for the interrelation of inclusive, active and participatory teaching and learning, over against a Physical Education, traditionally based on processes linked only to memorized methodology and mechanized and specific motor skills (e.g., technification and performance) (González-Víllora et al., 2009). Therefore, we look for methodological experiences that promote positive pedagogical practices with interventions based on teaching models (IM: *Instructional Models*) (Metzler, 2017) or based on practice (MsBP: *Models-Based Practice*) (Casey, 2014). These pedagogical models developed in a safe and contextualized way (Casey and MacPhail, 2018; González-Víllora et al., 2019) versus traditional decontextualized educational models (DI: *Direct Instruction*) (Sierra-Díaz et al., 2019) will be more motivating for students and will significantly improve the practice of physical-sports content, social and interpersonal relationships (Gil-Arias et al., 2017).

The Sport Education Model (SEM) (Hastie and Wallhead, 2016; Bessa et al., 2019; Kao, 2019; Luna et al., 2019; Siedentop et al., 2019) is among the most suitable pedagogical models (Iserbyt et al., 2016) to develop the affective-social dimension of students. This is a model (MsBP) whose purpose is that all students live authentic sports experiences (Siedentop et al., 2019). Likewise, the SEM (Siedentop, 1994) intends to develop competent, enthusiastic, and physically (Whitehead, 2010) and sportingly literate students (Kolovelonis and Goudas, 2018). Thus, United Nations Educational Scientific and Cultural Organization [UNESCO] (2015) reports that the practice of healthy and active sports activities in organized games and sports, such as those planned and developed in the SEM and instrumentalized in quality Physical Education, show a positive impact on psychosocial adjustment of students, as well as in their emotional, physical, and cognitive dimension. Therefore, it is a favorable pedagogical model for proactive social development, positive responsibility, and more equitable and inclusive learning (Farias et al., 2019).

In the same line, some systematic reviews conclude that through interventions based on the SEM, there are improvements in technical-tactical skills (physical and cognitive physical domain), social and emotional development (Evangelio et al., 2018; González-Víllora et al., 2018) and motivational aspects (Chu and Zhang, 2018). In addition, meta-analysis (e.g., Sierra-Díaz et al., 2019) confirms benefits in motivation toward physical and sports activity, belonging and social responsibility, autonomy, and organization. Recent SEM results show positive effects on motor behaviors (Pereira et al., 2015; Wahl-Alexander and Chomentowski, 2018; Araújo et al., 2019), technical-tactical skills (Farias et al., 2015) and activity, knowledge, and physical performance (Ward et al., 2017). Also, improvements in trait emotional intelligence and subjective well-being (Luna et al., 2019), motivation (Cuevas et al., 2016; Gil-Arias et al., 2017), social cohesion and social skills (Kao, 2019; Pan et al., 2019), attitudes toward violence, social responsibility, and friendly relations (Menéndez-Santurio and Fernández-Río, 2016), assertiveness (García-López and Gutiérrez, 2015), and social relations (Perlman, 2010) have been found. However, regarding the statistical analysis of the data, in most of these previous studies, the change/gain score through analysis of variance (ANOVA) (that is, posttest minus pretest) was used as a criterion group comparison. In this sense, some authors such as Pérez-González and Qualter (2018) recommend the use of analysis of covariance (ANCOVA) where the covariate is the baseline or pretest score, controlling for the possible effect of the pretest score on the results of the posttest. Criteria followed in the present study are in the same line as other authors (e.g., Menéndez-Santurio and Fernández-Río, 2016; Kao, 2019; Luna et al., 2019; Pan et al., 2019).

On the other hand, it is necessary to point out that there are stereotyped preconceptions and sports discrimination based on gender (Leaper, 2011; Parker and Curtner-Smith, 2012). Along these lines, some previous studies on interventions based on SEM have not shown differences in their impact, depending on gender, physical abilities (Araújo et al., 2019) and socio-emotional skills (Evangelio et al., 2018). On the contrary, other researches have shown differences in impact, depending on gender: some studies conclude that boys have greater improvements than girls in social interactions (Brock et al., 2009; Hastie et al., 2009), while other research confirms more significant improvements in girls than in boys in technical-tactical sports knowledge (Mesquita et al., 2012).

Accordingly to all this, the purpose of the current study was to evaluate the effects of a SEM-based intervention, compared to an intervention based on the Traditional Model of Direct Instruction (TM-DI), in adolescents on the variables: (1) social competence and (2) social acceptance among peers. Regarding the hypotheses, it was proposed that said intervention (based on the SEM) would improve the participants’ social competence (H1) and social acceptance among peers (H2). Finally, the impact of the intervention would not show differences, depending on gender (H3) in line with previous studies (Evangelio et al., 2018).

## Materials and Methods

### Design

A randomized experimental design was conducted with two repeated measures (pretest and posttest). The participants were randomly assigned to the experimental group (EG) and control group (CG) through a randomized controlled group trial.

### Participants

The total sample was composed of 114 adolescents, aged between 12 and 15 years (mean age (*M*) = 13.41 years; standard deviation (*SD*) = 0.81 years). Regarding the sociodemographic distribution of the sample: (a) by gender, 52% were girls, and 48% were boys; (b) by age, 35% were 12 years old, 48% 13 years old, 15% 14 years old, and 2% 15 years old. The differences in the two conditions (experimental and control groups) were not significant by age (χ^2^ = 1.08, *p* > 0.05) or by gender (χ^2^ = 1.14, *p* > 0.05).

**FIGURE 1:**
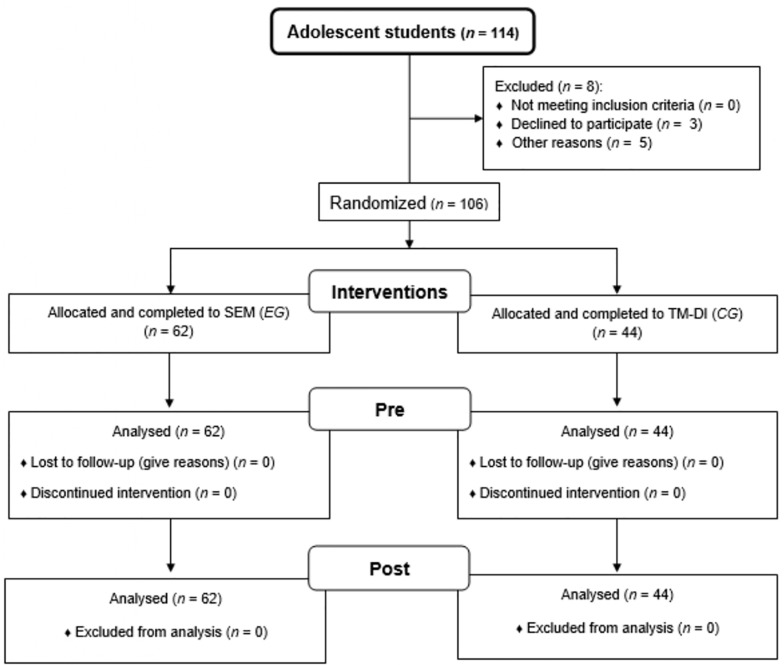
Participant flow diagram: interventions based on the sport education model (SEM) (*EG* = experimental group); traditional model of direct instruction (TM-DI) (*CG* = control group).

The criteria for inclusion (*n* = 106) in the study were: (1) regular attendance to school (≥80% of attendance) and (2) informed written consent from the parents (or legal guardian). The exclusion criteria (*n* = 8) were: (1) attend less than 80% of the educational intervention sessions (less than 13 sessions); (2) students with more than 30% truancy; (3) students with special educational needs; (4) students sanctioned for disciplinary reasons by the school; (5) did not obtain informed written consent from the parents (or legal guardian). The 106 participants who met the proposed criteria were randomly assigned to the EG (*n* = 62) or the CG (*n* = 44). Participant flow is displayed below (refer to Figure 1).

### Measures

In this investigation, two measures have been used to evaluate the proposed variables.

#### Adolescent Multidimensional Social Competence Questionnaire (AMSC-Q)

The Adolescent Multidimensional Social Competence Questionnaire (AMSC-Q) has been used to assess social competence. The instrument was validated in Spanish for its use with Spanish adolescents (Gómez-Ortiz et al., 2017). The AMSC-Q contains 26 Likert-type items scored on a scale from 1 to 7 (1 = *completely false*; 7 = *completely true*). This instrument measures five factors of social competence: cognitive reappraisal, social adjustment, prosocial behavior, perceived social efficacy, and normative adjustment. In the current study, the evidences of reliability are the following: cognitive reappraisal (α = 0.71; Ω = 0.70); social adjustment (α = 0.84; Ω = 0.83); prosocial behavior (α = 0.76; Ω = 0.72); perceived social efficacy (α = 0.80; Ω = 0.77); and normative adjustment (α = 0.79; Ω = 0.79).

#### Guess Who Questionnaire (GW4)

The Guess Who questionnaire (GW4) (Mavroveli et al., 2009); version adapted to Spanish by Losada et al. (2017) has been used to assess social acceptance among peers (SA). The GW4 is made up of four indicators of social behavior or attributes based on descriptors of habitual behavior patterns (kind; stalker; cooperator; leader). The descriptor *kind* indicates classmates who take into account the feelings of others, are friendly, and are generous with their things. The descriptor *stalker* is defined as classmates who often mess with other children, hit them, or behave unpleasantly for no reason. The descriptor *cooperator* indicates the classmates with whom they would form a group because they collaborate, participate, share, and respect others. The descriptor *leader* indicates peers who lead and encourage to keep going. Likewise, based on the results in each of the four indicators, a global score or Index of Social Acceptance (ISA) may be calculated, as a result of adding the nominations in the three prosocial domains and subsequently subtracting the score obtained in the antisocial domain. Previously, the nominations received in the four descriptors have to be transformed into percentages. In calculating the percentages for each descriptor, the account is taken of the total number of students in the class group, the total number of students responding in the class group, and the number of nominations allowed, which is unlimited, but at a minimum would be equal to one. The maximum number of nominations from one student is equal to the total number of students minus the nominating subject (Losada et al., 2017). In the current study, the evidence of reliability for the ISA is α = 0.76; Ω = 0.79.

### Procedure

The study design was developed in four periods. In the first period, the educational intervention was designed. Secondly, a pretest assessment (T1) was carried out in the experimental and control group, administering the assessment instruments with scheduled breaks to avoid student fatigue. The pretest evaluation was carried out as a group. The administration of the pretest evaluations was carried out by two members of the research team different from the teachers who implemented the program. In the third period, the educational intervention based on the SEM was applied in the EG, while in the CG, scheduled sessions of the Traditional Model of Direct Instruction (TM-DI) were developed. To minimize the effect of the experimenter on the results, the participating adolescents, and the teachers who applied each of the interventions were unaware of the hypotheses and objectives of the research team (single-blind procedure). Both happened during the Physical Education class on school hours. In the last period, at the end of the intervention, the posttest assessment (T2) was carried out in both groups, following the same rest procedure as in T1. Posttest evaluations were administered by two members of the research team different from the teachers who carried out the program. The posttest evaluation was developed in groups.

### Ethical Considerations

This study has been developed under the University of Castilla-La Mancha (UCLM) code of ethics, following international guidelines on experiments with human subjects described in the Nuremberg Code and the Declaration of Helsinki. The Management Team, the School Board, and the Teachers of the participating school authorized the investigation since it is an investigation framed within a public educational context. An informed written consent for the participating students was signed by a parent or legal guardian. Likewise, the requirements of ethical confidentiality were respected and guaranteed according to the voluntary and anonymous nature of the participants (ethical guidelines of the American Psychological Association [APA], 2019; Personal Data Protection Law of the Research Ethics Committee on Human Beings, CEISH).

### Educational Intervention

Two educational interventions were developed: in the experimental group, the intervention was based on the Sport Education model (SEM) (Siedentop et al., 2019) and in the control group, the intervention was based on the Traditional Model of Direct Instruction (TM-DI) (Metzler, 2017). Both were applied simultaneously during school hours by teachers specialized in Physical Education. One of the teachers developed the SEM-based intervention in the experimental group. This teacher has 15 years of teaching experience and 3 years applying the SEM in Physical Education (ecological validity). A different teacher applied a Traditional Model of Direct Instruction (TM-DI) in the control group. This teacher has 10 years of teaching experience, without previous SEM experience. They were carried out during 16 sessions of 55 min each, with a frequency of two sessions per week (refer to Table 1). A sport of split teams or net (Polskie ringo) was used for both groups of adolescents (Méndez-Giménez et al., 2011). This alternative sport, which was new for the participating students, is played in teams on a sports field divided in two by a central volleyball net. Players must throw, receive and pass a ring over the net, scoring when the ring falls on the field of the opposite team.

**TABLE 1:** Sequence of sessions and activities in the educational interventions.

Session	SEM (experimental group)	TM-DI (control group)
1	Theoretical explanation of SEM and Polskie ringo. Delivery of teaching material (folders; match records; reports; game rules; contingency contract, etc.).	Theoretical explanation of Polskie ringo (regulatory aspects).
2–3	Training and organization of teams (choice of thematic names, hymns/emblems, identifying colors, etc.). Designation of rotating responsibility roles. Self-construction of material by student art (e.g., Polskie ringo ring).	Organization of students individually or in pairs (no persistent work groups are formed). Presentation of sports equipment provided by the school. Technical development activities (pass, launch and reception I).
4–7	Warm-up and stretching with modified sports games, directed by the teacher and students (role of physical trainer). Activities (by teams and using rotating roles of responsibility) aimed at learning technical and tactical skills of Polskie ringo (pass, serve, reception, throwing, displacements). Reflective-comprehensive meetings (positive feedback; active listening; learning-error). Knowledge of rules through the real game (fair play = sport key element). Pre-season or training for the championship (educational competition).	Warm-up sessions led by the teacher. Activities to develop technical skills repetitively (pass and reception II). Technical development activities (serve). Technical development activities (throwing). Technical development activities (displacement). Completion with stretching exercises, led by the teacher. Also, the teacher instructs the students for the improvement of the movements developed.
8–14	Friendly team matches and educational competition (fair play) through a formal and regular league (Round Robin). Development of responsibility roles (e.g., referee, journalist, captain…). Use of real sports elements (minutes, interviews, etc.).	Sports warm-up. Tactical development activities 1 vs. 1. Tactical development activities 2 vs. 2. Tactical development activities 3 vs. 3. Simultaneous Polskie ringo games. Final stretching exercises led by the teacher.
15–16	Semi-final and final competition between classes. Event with final festival (organized by the role of committee): delivery of trophies, diplomas and medals (self-built materials). Summative or final evaluation.	Individual theoretical assessment of Polskie ringo (regulatory aspects). Individual practical evaluation of Polskie ringo (technical elements).

#### Characteristics of the Intervention Based on the Sport Education Model (SEM)

The intervention design was developed following precisely and adequately the structure of the SEM (Siedentop et al., 2019) and the recommendations made by Hastie and Casey (2014). The educational experience was organized as follows: (1) *season*: long-term teaching unit; (2) *affiliation* and/or *team membership*: development of group identity and interpersonal cooperation; (3) performance of *rotating responsibility roles* (e.g., referee, captain, physical trainer, journalists, festival committee): individual and shared decision-making; (4) *regular competition*: practice of technical-tactical knowledge; (5) *data recording*: information gathering and analysis of the learning process; (6) *culminating and festive event*: final objectives for all students in a festive and motivating way.

The intervention was implemented for around 2 months, in a public educational center, within a rural environment and with a medium socioeconomic level. Likewise, it was supervised by external researchers, consisting of: (a) personal and online communication to solve possible issues; (b) regular visits to the school; (c) analysis and weekly verification of the research process.

The selection and training of the teams were carried out randomly (to break present groups). The educational practice was developed in different academic classes of Secondary Education (3 experimental groups with 5 teams in each) setting a total of 15 mixed teams with a random distribution of each participant following the principle of homogeneity according to gender and level of motor ability (Burgueño et al., 2017). All students of each team were always assigned two roles: one common to all (player) and another specific to each student: (1) *captain-coach* (coordinator and mediator of the team, in addition to acting as a communicative link between teachers-students and vice versa); (2) *referee* (in charge of the functions of conciliation and fair play of sports practice, in addition to being responsible for compliance with the rules of the game and formalization of the minutes and match reports); (3) *journalist* (in charge of statistics, data recording, and managing digital communication with an informative sports blog previously created); (4) *physical trainer* (direction of previous sports warm-ups and responsible for the team’ sports equipment); and (5) *celebration organizing committee* (responsible for self-built materials, coordinator, and manager of final festive events). This educational strategy of the rotating role aims to encourage students to develop social skills such as empathy, providing them with different tasks and insights.

In short, this is a real and educational sport experience that aims to engage students with a motivating methodology focused on cooperative teaching and learning processes between internal groups as elements such as the development of a formal competition (regular league focused on fair and social game), self-construction of their own sports materials (such as the ring for the game, medals, trophies, or diplomas) (Méndez-Giménez et al., 2016) or the celebration of the final event, making the personal and social development of the adolescents more significant (Bessa et al., 2019).

#### Characteristics of the Intervention Based on the Traditional Model of Direct Instruction (TM-DI)

A teaching unit about Polskie ringo was designed and implemented according to a traditional methodology (Metzler, 2017; Pan et al., 2019). The methodological characteristics of Direct Instruction were: (a) teaching and learning process focused on an outstanding position of the teachers, favoring an expository and unidirectional communication to the students; (b) transmission of educational content by teachers without student intervention (only occasionally for demonstration by modeling); (c) assignment of tasks mostly centered on decisions made by teachers, where students play a passive role, that is, a teaching style where only the teacher directs and determines the tasks, objectives, evaluation, rhythm and learning time of the planned sessions and activities; (d) development of an educational experience, by students, with sports activities characterized by technical, memorial, and repetitive motor skills individually; (e) mass education with no individualization, using sports materials provided by the school; (f) learning of decontextualized sports fundamentals and without experiencing real sports experience, that is, characterized by a first orientation of skills where students practice sports learning in isolation.

### Statistical Analysis

Following collection, data were analyzed with the SPSS software, version 24.0 (IBM Corp., Armonk, NY, United States). First, the normality of the variables under study was calculated with the Kolmogorov-Smirnov test, all of them adjusting to the assumption of normality (analyses performed with a 95% confidence interval). Second, the evidence of reliability was calculated with the reliability coefficient of Cronbach’s alpha (α) and the McDonald’s omega coefficient (Ω). Third, to determine the effectiveness of the educational intervention, the following statistical analyses were performed: (1) multivariate analyses of variance (MANOVA) with the total pretest scores of the variables under study, to confirm possible starting (initial) differences between the participants of the EG and CG; (2) descriptive (*M* = mean; *SD* = standard deviation) and variance (ANOVA) analyses with each of the scores obtained for the instruments used during the pretest phase; (3) in order to show significant improvements between the experimental and control group, multivariate analyses of covariance (MANCOVA) were calculated on the set of variables investigated; (4) descriptive analyses, and covariance analyses (ANCOVA) with posttest scores; (5) finally, the effect size of the differences was calculated with partial square eta (μ^2^) following four statistical ranges (Tabachnick and Fidell, 2007): 0–0.009, *negligible*; 0.010–0.089, *low-effect size*; 0.090–0.249, *medium-effect size*; and > 0.250, *big-effect size*.

## Results

The pretest MANOVA results did not reveal statistically significant differences between the groups prior to the intervention, Wilks’ Lambda, Λ = 0.491; *F*(9, 97) = 0.572; *p* = 0.273, with a low effect size (μ^2^ = 0.028; *r* = 0.04).

### Pretest Analysis

The results of ANOVA in the pretest phase (refer to Table 2) showed that before starting the intervention, there were no statistically significant differences in any of the study variables.

**TABLE 2:** Mean, standard deviation, analysis of variance, analysis of covariance, and effect size for differences in means (partial square eta) in the variables under study (social competence and peer social acceptance) in the experimental and control groups.

	Pretest				ANOVA			Posttest				ANCOVA		
	EG		GC					EG		CG				
	*M*	*SD*	*M*	*SD*	*F*	*p*	μ^2^	*M*	*SD*	*M*	*SD*	*F*	*p*	μ^2^
**SC**														
Cognitive reappraisal	4.68	1.35	4.71	1.40	0.453	0.915	0.002	4.72	1.40	4.68	1.42	0.454	0.243	0.010
Social adjustment	5.48	1.41	5.53	4.58	1.231	0.534	0.003	6.03	1.56	5.41	1.52	2.731	0.011	0.064
Prosocial behavior	5.45	1.38	5.67	1.31	0.915	0.246	0.004	6.07	1.53	5.72	1.48	3.447	0.002	0.078
Perceived social efficacy	5.37	1.44	5.41	1.65	1.472	0.717	0.003	6.09	1.48	5.32	1.72	4.315	0.008	0.072
Normative adjustment	5.59	1.51	5.54	1.39	0.882	0.766	0.002	5.61	1.48	5.50	1.42	0.744	0.816	0.009
**SA**														
Kind	0.31	0.10	0.32	0.14	0.617	1.014	0.009	0.34	0.11	0.30	0.15	1.126	0.414	0.011
Stalker	0.27	0.18	0.25	0.20	0.074	0.879	0.007	0.25	0.13	0.26	0.19	0.933	0.736	0.001
Cooperator	0.28	0.15	0.30	0.12	1.011	0.731	0.003	0.37	0.12	0.29	0.11	2.732	0.008	0.027
Leader	0.16	0.20	0.19	0.18	0.873	0.512	0.004	0.19	0.19	0.16	0.21	0.899	0.122	0.007
Index of social acceptance	0.77	0.48	0.81	0.41	0.342	0.873	0.002	0.87	0.45	0.75	0.40	3.715	0.010	0.045

*SC, social competence; SA, peer social acceptance; μ^2^ = eta square effect size; EG, experimental group (n = 62); CG, control group (n = 44); M, mean; SD, standard deviation.*

### Posttest Analysis

The results for the pretest-posttest MANCOVA did not reveal statistically significant differences between the two conditions, Wilks’ Lambda, Λ = 0.862; *F*(9, 97) = 1.661; *p* = 0.187, with a low-effect size (μ^2^ = 0.081; *r* = 0.10).

#### Effects on Social Competence

**FIGURE 2:**
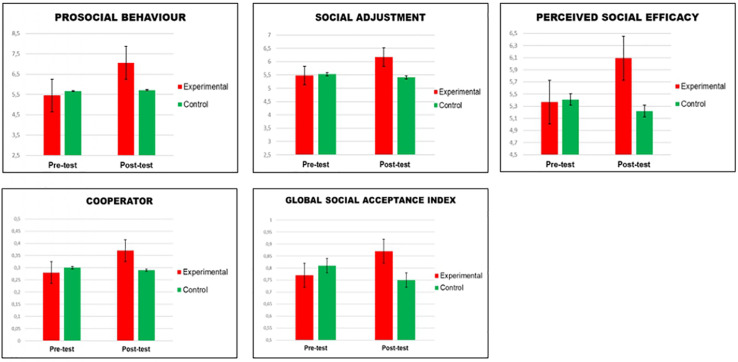
Statistically significant effects of SEM (pretest-posttest) in experimental group and control group.

After performing ANCOVA in the posttest phase (refer to Table 2), the results confirmed, in favor of the EG, significant improvements in: social adjustment, with a low-effect size (μ^2^ = 0.064); prosocial behavior, with a low-effect size (μ^2^ = 0.078); perceived social efficacy, with a low-effect size (μ^2^ = 0.072) (refer to Figure 2). However, no significant differences were confirmed in the other two factors, cognitive reappraisal and normative adjustment.

#### Effects on Social Acceptance Among Peers

The results in the ANCOVA in the posttest phase (refer to Table 2) showed significant improvements in the cooperator factor with a low-effect size (μ^2^ = 0.027) in favor of the EG. Likewise, the results showed significant improvements in favor of the experimental group in the global index of social acceptance among peers of this variable, with a low-effect size (μ^2^ = 0.045) (refer to Figure 2). However, there were no significant differences in the other indicators: kind, stalker, and leader.

#### Effects on Gender

The results of ANOVA for the pretest phase showed that before beginning the intervention, there were no statistically significant differences, depending on gender, in any of the study variables. Similarly, the results in the ANCOVA for the posttest phase did not show differential effects between boys and girls in any of the study variables.

## Discussion

The current study evaluated the effects of an intervention based on the SEM, compared to an intervention based on the TM-DI, on social competence and social acceptance among adolescents. It is necessary to highlight that recent studies raise the need to continue examining the impact of SEM-based interventions in adolescents (Evangelio et al., 2018; Bessa et al., 2019; Sierra-Díaz et al., 2019).

In general, the results showed statistically significant improvements, in favor of the experimental group (SEM) compared to the control group (MT-ID): (1) in some indicators of social competence; social adjustment, prosocial behavior, and perceived social efficacy. However, no significant improvements in cognitive reappraisal and normative adjustment were confirmed; (2) improvements in social acceptance among peers; specifically, in the *cooperator* factor and in the global index of social acceptance among peers. Although no improvements were found in the factors: *kind*, *stalker*, and *leader*.

First, the results showed significant improvements in some indicators of social competence. Therefore, Hypothesis 1 is partially confirmed. Specifically, the positive impact has focused on (1) social adjustment, that is, the degree to which an adolescent engages in socially competent behaviors, whose purpose is social acceptance; (2) prosocial behavior, defined as voluntary behaviors whose purpose is to benefit others (sharing, caring, comforting or helping); and (3) perceived social efficacy, or the subjective perception of effectiveness in social interactions. These results converge with other previous research papers that analyze the effectiveness of interventions based on the SEM in some socio-emotional variables (Perlman, 2010; García-López and Gutiérrez, 2015; Menéndez-Santurio and Fernández-Río, 2016; Méndez-Giménez et al., 2016; González-Víllora et al., 2018; Bessa et al., 2019; Kao, 2019; Luna et al., 2019; Pan et al., 2019). The results of these previous investigations confirm in the variables under consideration, effect sizes between low and moderate, in line with those obtained in this study.

Secondly, the results show some significant improvements in the variable social acceptance among peers, specifically in the *cooperator* indicator and the global social acceptance score or *Index of Social Acceptance* (ISA). Hypothesis 2 is partially confirmed. These findings are in line with other research that has shown the positive impact of MbBP-SEM on the social relationship (García-López and Gutiérrez, 2015; Menéndez-Santurio and Fernández-Río, 2016; Kao, 2019). The conclusions of these previous investigations in the variables being considered point to effect sizes between low and moderate, in congruence with those obtained in the current study. As we can see, the effects obtained in social competence are higher than those obtained in social acceptance. In this sense, the nuclear aspects of the MED are probably closer to the development of helping behaviors toward others, that is, toward efficacy in social interaction (Gómez-Ortiz et al., 2019) than to the social acceptance within the group.

Third, the results did not confirm differential effects between boys and girls in any of the study variables. These results are consistent with those obtained in some systematic reviews that conclude that the development of interventions based on the SEM has shown improvements in the participants in empathy, assertiveness, and fair play, regardless of gender (e.g., Evangelio et al., 2018). However, it is necessary to deepen this line of research. On the one hand, numerous studies conclude that girls have higher social skills scores than boys; while, boys have higher levels of rejection compared to girls (Bandura et al., 2006) due to the contradictory and inconclusive results concerning the different impact of SEM-based interventions on boys and girls (Evangelio et al., 2018).

These positive results used in the social competence indicators probably favor adaptive interpersonal relationships (Eisenberg et al., 2006). In the same way, this improvement in social interaction among peers may be influenced by the intrapersonal and interpersonal emotional regulation strategies underlying the successful adaptation of adolescents to the requirements in social relationships (Mestre-Navas and Guil, 2012). Also, social acceptance is positively related to the behaviors that help to follow the rules of collective games and be actively involved in adaptive interactions with their peers (Trianes et al., 1999).

Another possible explanation of these results could be the methodology used in the intervention, based on cooperative learning and encouraging the motivation of the participants (Casey, 2014; Metzler, 2017; González-Víllora et al., 2018; Gil-Madrona et al., 2019; Sierra-Díaz et al., 2019) as well as, for the improvement of assertiveness, cooperation, autonomy and positive communication among peers (García-López and Gutiérrez, 2015). Also, the intervention aims to favor team sport and increase the responsibility of each participant in achieving a common goal (Méndez-Giménez et al., 2011, 2016; Kolovelonis and Goudas, 2018). Authors such as Washington et al. (2001) consider sport as a fundamental tool for social transformation, which will allow the promotion of cooperative learning through the assignment and distribution of responsibility roles (Siedentop et al., 2019) and will foster enthusiasm and enjoyment for an educational and cooperative sports practice (Iserbyt et al., 2016; Evangelio et al., 2018; Gil-Madrona et al., 2019; Pan et al., 2019).

Likewise, it is necessary to highlight that adolescence is a crucial stage for the development of socio-emotional competencies, since adolescents experience this stage with constant and typical maturational and emotional transitions of greater social difficulty (Gómez-Ortiz et al., 2017, 2019). In this sense, identifying, controlling and managing socio-emotional competences contribute to optimize teaching and learning processes, strengthening social interaction among adolescents (Del Prette and Del Prette, 2005; Ang and Penney, 2013; Gómez-Ortiz et al., 2017; Losada et al., 2017; Cañabate et al., 2018; Evangelio et al., 2018) and can favor an efficient evaluation of the educational practice made by the teachers (Lee et al., 2019).

### Limitations and Future Directions

The current study had some limitations. First, it would have been necessary to carry out a follow-up evaluation of the intervention to analyze the long-term effect on the variables studied. Secondly, it would be necessary to use instruments completed by teachers or families that improve the assessment of the variables studied. Thirdly, it would be necessary to include a session analysis procedure in order to assess whether teachers followed the main principles of the model (formative evaluation) (Práxedes et al., 2019). Fourth, it is necessary to point out that the results obtained in Hypothesis 3 should be interpreted with great caution due to the sample size. However, studies that analyze this aspect should be carried out (Evangelio et al., 2018). Fifth, in terms of minimizing the effect of the experimenter, it would have been necessary for the posttest evaluation to use the balancing procedure of the members of the research team that administered the tests in each of the experimental conditions.

One of the most relevant contributions of the present study was the use of statistical analysis through ANCOVA, which evaluates the effectiveness of educational interventions as opposed to other statistical methods, such as the use of ANOVA, which evaluates changes or gains. These findings, through appropriate statistical procedures, could enrich research concerning SEM (e.g., Menéndez-Santurio and Fernández-Río, 2016; Kao, 2019; Luna et al., 2019; Pan et al., 2019).

Finally, it is necessary to highlight the difficulties in following the recommendations of the SEM in teaching sessions (Hastie and Casey, 2014; Siedentop et al., 2019). On the other hand, future lines of research could be: (1) to increase the sample and diversify the socio-cultural environment of adolescents; (2) assessment of the variables involved in the improvements obtained through these interventions, such as emotional regulation strategies (intrapersonal and interpersonal).

## Conclusion

These significant results are likely due, as some research suggests, to the positive synergy among physical activity developed in positive environments (Mayfield et al., 2017; Shields et al., 2018; Escalié et al., 2019; Gil-Madrona et al., 2019) such as quality Physical Education (Association for Physical Education [afPE], 2015; United Nations Educational Scientific and Cultural Organization [UNESCO], 2015; Shields et al., 2018) with affective and/or psychosocial factors (Kao, 2019; Pate and Dowda, 2019; Sierra-Díaz et al., 2019). Said context will facilitate in students better pedagogical strategies (Girard et al., 2019) that make socio-emotional learning more positive (Brinkley et al., 2017; Mayfield et al., 2017; Cronin et al., 2018) and therefore, improve their educational experience (Kohl and Cook, 2013; Sierra-Díaz et al., 2019).

It is relevant to note that these findings suggest that when classes are developed with a quality Physical Education (United Nations Educational Scientific and Cultural Organization [UNESCO], 2015) using effective pedagogical models such as the SEM (Franco et al., 2017), students show high levels of positive emotions and social skills favoring peer interactions, clear evidence of cooperation, and human relationships that promote prosocial coexistence (Cañabate et al., 2018).

## Data Availability Statement

All datasets generated for this study are included in the article/supplementary material.

## Ethics Statement

Ethical review and approval was not required for the study on human participants in accordance with the local legislation and institutional requirements. Written informed consent to participate in this study was provided by the participants’ legal guardian/next of kin.

## Author Contributions

PL and JC conceived and designed the work. PL and JC were responsible for the design of the educational intervention, implemented by JG. PL, DR-R, LL, and JC collected the data and drafted the manuscript. PL, JG, LL, and JC were responsible for the data analysis and interpretation. PL, JG, DR-R, and JC were responsible for critical revision of the manuscript. PL, JG, DR-R, LL, and JC approved the final version of the manuscript to be published. All authors made substantial contributions to the work.

## Conflict of Interest

The authors declare that the research was conducted in the absence of any commercial or financial relationships that could be construed as a potential conflict of interest.

## References

[B1] American Psychological Association [APA] (2019). *Ethical Principles of Psychologists and Code of Conduct.* Available online at: https://www.apa.org/ethics/code/index (accessed May 27, 2020).

[B2] Ang S. C. Penney D. (2013). Promoting social and emotional learning outcomes in physical education: insights from a school-based research project in Singapore. *Asia Pac. J. Health Sport Phys. Educ.* 4 267–286. 10.1080/18377122.2013.836768

[B3] Araújo R. Hastie P. Lohse K. R. Bessa C. Mesquita I. (2019). The long-term development of volleyball game play performance using sport education and the step-game-approach model. *Eur. Phys. Educ. Rev.* 25 311–326. 10.1177/1356336X17730307

[B4] Association for Physical Education [afPE] (2015). *Health Position Paper.* Available online at: https://www.afpe.org.uk/images/stories/afPE_Health_Position_Paper_Web_Version.pdf (accessed November 24, 2019).

[B5] Bailey R. (2006). Physical education and sport in schools: a review of benefits and outcomes. *J. Sch. Health* 76 397–401. 10.1111/j.1746-1561.2006.00132.x 16978162

[B6] Bandura M. Silva S. Cordeiro L. Pereira Z. Del Prette A. (2006). Habilidades sociais evariáveis sociodemográficas em estudantes do ensino fundamental. *Psicol. Em. Estudo* 11 541–549. 10.1590/S1413-73722006000300010

[B7] Bessa C. Hastie P. Araújo R. Mesquita I. (2019). What do we know about the development of personal and social skills within the sport education model: a systematic review. *J. Sports Sci. Med.* 18 812–829. 31827367 PMC6873138

[B8] Bisquerra R. Pérez-González J. C. García E. (2015). *Inteligencia Emocional En Educación [Emotional Intelligence In Education].* Madrid: Síntesis.

[B9] Brinkley A. McDermott H. Munir F. (2017). What benefits does team sport hold for the workplace? A systematic review. *J. Sports Sci.* 35 136–148. 10.1080/02640414.2016.1158852 26979430

[B10] Brock S. J. Rovegno I. Oliver K. L. (2009). The influence of student status on student interactions and experiences during a sport education unit. *Phys. Educ. Sport Pedagogy* 14 355–375. 10.1080/17408980802400494

[B11] Burgueño R. Medina-Casaubón J. Morales-Ortiz E. Cueto-Martín B. Sánchez-Gallardo I. (2017). Sport education versus traditional teaching: influence on motivational regulation in high school students. *Cuad. Psicol. Deporte* 17 87–98.

[B12] Cañabate D. Martínez G. Rodríguez D. Colomer J. (2018). Analysing emotions and social skills in physical education. *Sustainability* 10:1585 10.3390/su10051585

[B13] Cappadocia M. C. Weiss J. A. (2011). Review of social skills training groups for youth with asperger syndrome and high functioning autism. *Res. Autism Spectr. Disord.* 5 70–78. 10.1016/j.rasd.2010.04.001

[B14] Casey A. (2014). Models-based practice: great white hope or white elephant? *Phys. Educ. Sport Pedagogy* 19 18–34. 10.1080/17408989.2012.726977

[B15] Casey A. MacPhail A. (2018). Adopting a models-based approach to teaching physical education. *Phys. Educ. Sport Pedagogy* 23 294–310. 10.1080/17408989.2018.1429588

[B16] Cecchini-Estrada J. A. González González-Mesa C. Méndez-Giménez A. Fernández-Río J. (2011). Achievement goals, social goals, and motivational regulations in physical education settings. *Psicothema* 23 51–57. 21266142

[B17] Chu T. L. Zhang T. (2018). Motivational processes in sport education programs among high school students: a systematic review. *Eur. Phys. Educ. Rev.* 24 372–394. 10.1177/1356336X17751231

[B18] Cronin L. D. Allen J. Mulvenna C. Russell P. (2018). An investigation of the relationships between the teaching climate, students’ perceived life skills development and well-being within physical education. *Phys. Educ. Sport Pedagogy* 23 181–196. 10.1080/17408989.2017.1371684

[B19] Cuevas R. García-López L. M. Serra-Olivares J. (2016). Sport education model and self-determination theory: an intervention in secondary school children. *Kinesiology* 48 30–38. 10.26582/k.48.1.15

[B20] Del Prette Z. A. P. Del Prette A. (2005). *Psicologia Das Habilidades Sociais Na Infancia: Teoria e Prática.* Petrópolis: Vozes.

[B21] Dirks M. A. Treat T. A. Weersing V. R. (2007). Integrating theoretical, measurement, and intervention models of youth social competence. *Clin. Psychol. Rev.* 27 327–347. 10.1016/j.cpr.2006.11.002 17270330

[B22] Domitrovich C. E. Durlak J. A. Staley K. C. Weissberg R. P. (2017). Social-emotional competence: an essential factor for promoting positive adjustment and reducing risk in school children. *Child Dev.* 88 408–416. 10.1111/cdev.12739 28213889

[B23] Eisenberg N. Fabes R. A. Spinrad T. L. (2006). “Handbook of childpsychology: social, emotional, and personality development,” in *Prosocial Development*, ed. Eisenberg N. (Hoboken, NJ: Wiley), 646–718. 10.1002/9780470147658.chpsy0311

[B24] Eldar E. (2008). Educating through the physical—behavioral interpretation. *Phys. Educ. Sport Pedagogy* 13 215–229. 10.1080/17408980701345741

[B25] Elijah D. W. Madeira J. M. (2013). Efeitos da intervenção social cognitiva para a melhoria da competência social e do sucesso escolar em alunos de escola primária inglesa: estudo de caso. *Saber Educ.* 18 94–105. 10.17346/se.vol18.54

[B26] Elliot A. J. Gable S. L. Mapes R. R. (2006). Approach and avoidance motivation in the social domain. *Pers. Soc. Psychol. Bull.* 32 378–391. 10.1177/0146167205282153 16455864

[B27] Escalié G. Recoules N. Chaliès S. Legrain P. (2019). Helping students build competences in physical education: theoretical proposals and illustrations. *Sport Educ. Soc.* 24 390–403. 10.1080/13573322.2017.1397507

[B28] Evangelio C. Sierra-Díaz M. J. González-Víllora S. Fernández-Rio F. J. (2018). The sport education model in elementary and secondary education: a systematic review. *Movement* 24 931–946. 10.22456/1982-8918.81689

[B29] Farias C. Wallhead T. Mesquita I. (2019). ‘The project changed my life’: sport education’s transformative potential on student physical literacy. *Res. Q. Exerc. Sport* 91 263–278. 10.1080/02701367.2019.1661948 31718525

[B30] Farias C. F. Mesquita I. R. Hastie P. A. (2015). Game performance and understanding within a hybrid sport education season. *J. Teach. Phys. Educ.* 34 363–383. 10.1123/jtpe.2013-0149

[B31] Franco M. G. Beja M. J. Candeias A. Santos N. (2017). Emotion understanding, social competence and school achievement in children from primary school in Portugal. *Front. Psychol.* 8:1376. 10.3389/fpsyg.2017.01376 28861014 PMC5559500

[B32] García-López L. M. Gutiérrez D. (2015). The effects of a sport education season on empathy and assertiveness. *Phys. Educ. Sport Pedagogy* 20 1–16. 10.1080/17408989.2013.780592

[B33] Garn A. McCaughtry N. Shen B. Martin J. Fahlman M. (2011). Social goals in urban physical education: relationships with effort and disruptive behavior. *J. Teach. Phys. Educ.* 30 410–423. 10.1123/jtpe.30.4.410

[B34] Gil-Arias A. Harvey S. Cárceles A. Práxedes A. Del Villar F. (2017). Impact of a hybrid TGfU-Sport education unit on student motivation in physical education. *PLoS One* 12:e0179876. 10.1371/journal.pone.0179876 28658267 PMC5489183

[B35] Gil-Madrona P. Gutiérrez-Marín E. C. Cupani M. Samalot-Rivera A. Díaz-Suárez A. López-Sánchez G. F. (2019). The effects of an appropriate behavior program on elementary school children social skills development in physical education. *Front. Psychol.* 10:1998. 10.3389/fpsyg.2019.01998 31632310 PMC6786239

[B36] Girard S. St-Amand J. Chouinard R. (2019). Motivational climate in physical education, achievement motivation, and physical activity: a latent interaction model. *J. Teach. Phys. Educ.* 38 305–315. 10.1123/jtpe.2018-0163

[B37] Gómez-Ortiz O. Romera E. M. Ortega-Ruiz R. (2017). Multidimensionality of social competence: measurement of the construct and its relationship with bullying roles. *Rev. Psicodidáct. Engl. Edn.* 22 37–44. 10.1387/RevPsicodidact.15702

[B38] Gómez-Ortiz O. Romera E. M. Ortega-Ruiz R. Herrera M. O’Higgins Norman J. (2019). Multidimensional social competence in research on bullying involvement: a cross-cultural study. *Behav. Psychol.* 27 217–238.

[B39] González-Víllora S. Evangelio C. Sierra-Díaz J. Fernández-Río J. (2018). Hybridizing pedagogical models: a systematic review. *Eur. Phys. Educ. Rev.* 25 1056–1074. 10.1177/1356336X18797363

[B40] González-Víllora S. García-López L. M. Contreras-Jordan O. R. Sánchez-Mora Moreno D. (2009). The concept of sport initiation nowadays. *Retos* 15 14–20.

[B41] González-Víllora S. Sierra-Díaz M. J. Pastor-Vicedo J. C. Contreras-Jordán O. R. (2019). The way to increase the motor and sport competence among children: the contextualized sport alphabetization model. *Front. Physiol.* 10:569. 10.3389/fphys.2019.00569 31156456 PMC6532438

[B42] Greenberg M. T. Weissberg R. P. O’Brien M. U. Zins J. E. Fredericks L. Resnik H. (2003). Enhancing school-based prevention and youth development through coordinated social, emotional, and academic learning. *Am. Psychol.* 58 466–474. 10.1037/0003-066x.58.6-7.466 12971193

[B43] Gresham F. M. (1988). “Social skills: conceptual and applied aspects of assessment, training, and social validation,” in *Handbook of Behavior Therapy In Education*, eds Witt J. C. Elliott S. N. Gresham F. M. (New York, NY: Plenum Press), 523–546. 10.1007/978-1-4613-0905-5_20

[B44] Hastie P. A. Casey A. (2014). Fidelity in models-based practice research in sport pedagogy: a guide for future investigations. *J. Teach. Phys. Educ.* 33 422–431. 10.1123/jtpe.2013-0141

[B45] Hastie P. A. Sinelnikov O. A. Guarino A. J. (2009). The development of skill and tactical competencies during a season of badminton. *Eur. J. Sport Sci.* 9 133–140. 10.1080/17461390802542564

[B46] Hastie P. A. Wallhead T. (2016). Models-based practice in physical education: the case for sport education. *J. Teach. Phys. Educ.* 35 390–399. 10.1123/jtpe.2016-0092

[B47] Hellison D. (2011). *Teaching Personal and Social Responsibility Through Physical Activity.* Champaign, IL: Human Kinetics Publishers.

[B48] Iserbyt P. Ward P. Martens J. (2016). The influence of content knowledge on teaching and learning in traditional and sport education contexts: an exploratory study. *Phys. Educ. Sport Pedagogy* 21 539–556. 10.1080/17408989.2015.1050662

[B49] Kao C. C. (2019). Development of team cohesion and sustained collaboration skills with the sport education model. *Sustainability* 11:2348 10.3390/su11082348

[B50] Kohl H. W. Cook H. D. (eds) (2013). *Educating the Student Body: Taking Physical Activity And Physical Education To School.* Washington, DC: National Academies Press, 10.17226/18314 24851299

[B51] Kolovelonis A. Goudas M. (2018). The relation of physical self-perceptions of competence, goal orientation, and optimism with students’ performance calibration in physical education. *Learn. Individ. Differ.* 61 77–86. 10.1016/j.lindif.2017.11.013

[B52] Leaper C. (2011). Research in developmental psychology on gender and relationships: reflections on the past and looking into the future. *Br. J. Dev. Psychol.* 29 347–356. 10.1111/j.2044-835X.2011.02035.x 21592154

[B53] Lee Y. H. Kwon H. H. Richards K. A. (2019). Emotional intelligence, unpleasant emotions, emotional exhaustion, and job satisfaction in physical education teaching. *J. Teach. Phys. Educ.* 38 262–270. 10.1123/jtpe.2018-0177

[B54] Losada L. (2018). Reflection and construction of knowledge concerning social skills and social competence. *Rev. Caribeña Investig. Educ.* 2 7–22. 10.32541/recie

[B55] Losada L. Cejudo J. Benito-Moreno S. Pérez-González J. C. (2017). Guess Who 4 sociometric questionnaire as screening of social competence in elementary education. *Univ. Psychol.* 16 1 10.11144/Javeriana.upsy16-4.cscs

[B56] Luna P. Guerrero J. Cejudo J. (2019). Improving adolescents’ subjective well-being, trait emotional intelligence and social anxiety through a programme based on the sport education model. *Int. J. Environ. Res. Public. Health* 16:1821. 10.3390/ijerph16101821 31126004 PMC6571931

[B57] Mavroveli S. Petrides K. V. Sangareau Y. Furnham A. (2009). Exploring the relationships between trait emotional intelligence and objective socio-emotional outcomes in childhood. *Br. J. Educ. Psychol.* 79 259–272. 10.1348/000709908X368848 18950549

[B58] Mayfield C. A. Child S. Weaver R. G. Zarrett N. Beets M. W. Moore J. B. (2017). Effectiveness of a playground intervention for antisocial, prosocial, and physical activity behaviors. *J. Sch. Health* 87 338–345. 10.1111/josh.12506 28382669

[B59] Méndez-Giménez A. Cecchini-Estrada J. A. Fernández-Río J. (2018). A multi-theoretical approach of the students’ motivational profiles in physical education: achievement and social goals. *Psicothema* 30 401–407. 10.7334/psicothema2018.88 30353841

[B60] Méndez-Giménez A. Fernández-Río J. García-López L. M. González-Víllora S. Gutiérrez D. Martínez J. (2011). *Modelos Actuales De Iniciación Deportiva: Unidades Didácticas Sobre Juegos Y Deportes De Cancha Dividida.* Sevilla: Wanceulen Editorial Deportiva.

[B61] Méndez-Giménez A. Martínez de Ojeda Pérez D. Valverde-Pérez J. J. (2016). Students and teachers assessment of conventional and self-made material: longitudinal crossover study in physical education. *Retos* 30 20–25.

[B62] Menéndez-Santurio J. I. Fernández-Río J. (2016). Violence, responsibility, friendship and basic psychological needs: effects of a sport education and teaching for personal and social responsibility program. *Rev. Psicodidáct.* 21:15269 10.1387/RevPsicodidact.15269

[B63] Mesquita I. Farias C. Hastie P. (2012). The impact of a hybrid sport education–invasion games competence model soccer unit on students’ decision making, skill execution and overall game performance. *Eur. Phys. Educ. Rev.* 18 205–219. 10.1177/1356336X12440027

[B64] Mestre-Navas J. M. Guil R. (2012). *La Regulación De Las Emociones: Una Vía A La Adaptación Personal Y Social.* Madrid: Pirámide.

[B65] Metzler M. W. (2017). *Instructional Models for Physical Education*, 3th Edn, London: Routledge.

[B66] Mitchell D. Hutchinson C. J. (2003). Using graphic organizers to develop the cognitive domain in physical education. *J. Phys. Educ. Recreat. Dance* 74 42–47. 10.1080/07303084.2003.10608519

[B67] Pan Y. H. Huang C. H. Lee I. S. Hsu W. T. (2019). Comparison of learning effects of merging TPSR respectively with sport education and traditional teaching model in high school physical education classes. *Sustainability* 11:2057 10.3390/su11072057

[B68] Parker M. B. Curtner-Smith M. D. (2012). Sport education: a panacea for hegemonic masculinity in physical education or more of the same? *Sport Educ. Soc.* 17 479–496. 10.1080/13573322.2011.608945

[B69] Pate R. R. Dowda M. (2019). Raising an active and healthy generation: a comprehensive public health initiative. *Exerc. Sport Sci. Rev.* 47 3–14. 10.1249/JES.0000000000000171 30334849

[B70] Payton J. W. Weissberg R. P. Durlak J. A. Dymnicki A. B. Taylor R. D. Schellinger K. B. (2008). *The Positive Impact Of Social And Emotional Learning For Kindergarten To Eighth-Grade Students: Findings From Three Scientific Reviews.* Chicago: Collaborative for Academic, Social, and Emotional Learning.

[B71] Pereira J. Hastie P. Araújo R. Farias C. Rolim R. Mesquita I. (2015). A comparative study of students’ track and field technical performance in sport education and in a direct instruction approach. *J. Sports Sci. Med.* 14 118–127. 25729299 PMC4306763

[B72] Pérez-González J. C. Qualter P. (2018). “Emotional intelligence and emotional education in the school years,” in *An Introduction to Emotional Intelligence*, eds Dacree Pool L. Qualter P. (Chichester: Wiley), 81–104.

[B73] Perlman D. (2010). Change in affect and needs satisfaction for amotivated students within the sport education model. *J. Teach. Phys. Educ.* 29 433–445. 10.1123/jtpe.29.4.433

[B74] Práxedes A. Del Villar, Álvarez F. Moreno A. Gil-Arias A. Davids K. (2019). Effects of a nonlinear pedagogy intervention programme on the emergent tactical behaviours of youth footballers. *Phys. Educ. Sport Pedagogy* 24 332–343. 10.1080/17408989.2019.1580689

[B75] Santos A. J. Peceguina I. Daniel J. R. Shin N. Vaughn B. E. (2013). Social competence in preschool children: replication of results and clarification of a hierarchical measurement model. *Soc. Dev.* 22 163–179. 10.1111/sode.12007

[B76] Shields D. L. Funk C. D. Bredemeier B. L. (2018). Relationships among moral and contesting variables and prosocial and antisocial behavior in sport. *J. Moral Educ.* 47 17–33. 10.1080/03057240.2017.1350149

[B77] Siedentop D. (1994). *Sport Education: Quality PE Through Positive Sport Experiences.* Champaign, IL: Human Kinetics Publishers.

[B78] Siedentop D. L. Hastie P. Mars H. V. D. (2019). *Complete Guide To Sport Education.* Champaign, IL: Human Kinetics.

[B79] Sierra-Díaz M. J. González-Víllora S. Pastor-Vicedo J. C. López-Sánchez G. F. (2019). Can we motivate students to practice physical activities and sports through models-based practice? A systematic review and meta-analysis of psychosocial factors related to physical education. *Front. Psychol.* 10:2115. 10.3389/fpsyg.2019.02115 31649571 PMC6795761

[B80] Tabachnick B. G. Fidell L. S. (2007). *Using Multivariate Statistics*, 5th Edn, New York, NY: Allyn and Bacon.

[B81] Taylor R. D. Oberle E. Durlak J. A. Weissberg R. P. (2017). Promoting positive youth development through school-based social and emotional learning interventions: a meta-analysis of follow-up effects. *Child Dev.* 88 1156–1171. 10.1111/cdev.12864 28685826

[B82] Trianes M. V. De la Morena L. Muñoz A. (1999). *Relaciones Sociales Y Prevención De La Inadaptación Social Y Escolar.* Málaga: Ediciones Aljibe.

[B83] United Nations Educational Scientific and Cultural Organization [UNESCO] (2015). *Quality Physical Education (QPE): guidelines for policy makers. UNESCO Digital Library.* Available online at: https://unesdoc.unesco.org/ark:/48223/pf0000231101 (accessed January 23, 2020).

[B84] Unlu H. Karahan M. Aydos L. Oner M. (2011). A comparative study: the attitudes of Turkish and foreign male students to the physical education lesson. *Croat. J. Educ.* 13 169–187.

[B85] Wahl-Alexander Z. Chomentowski P. (2018). Impact of a university physical conditioning sport education season on students’ fitness levels. *Health Educ. J.* 77 828–836. 10.1177/0017896918776340

[B86] Ward J. K. Hastie P. A. Wadsworth D. D. Foote S. Brock S. J. Hollett N. (2017). A sport education fitness season’s impact on students’ fitness levels, knowledge, and in-class physical activity. *Res. Q. Exerc. Sport* 88 346–351. 10.1080/02701367.2017.1321100 28524725

[B87] Washington R. L. Bernhardt D. T. Gomez J. Johnson M. D. Martin T. J. Rowland T. W. (2001). Organized sports for children and preadolescents. *Pediatrics* 107 1459–1462. 10.1542/peds.107.6.1459 11389277

[B88] Whitehead M. (2010). *Physical Literacy: Throughout the Lifecourse.* London: Routledge.

[B89] World Health Organization [WHO] (2016). *Fiscal Policies For Diet And The Prevention Of Noncommunicable Diseases.* Geneva: WHO.

[B90] Zhang F. You Z. Fan C. Gao C. Cohen R. Hsueh Y. (2014). Friendship quality, social preference, proximity prestige, and self-perceived social competence: interactive influences on children’s loneliness. *J. Sch. Psychol.* 52 511–526. 10.1016/j.jsp.2014.06.001 25267172

